# Green Synthesis of Vanadium Dioxide Nanoparticles by *Shewanella* sp. Strain HN-41

**DOI:** 10.4014/jmb.2502.02051

**Published:** 2025-05-26

**Authors:** Yongseok Ko, Saehyun Kang, Youri Yang, Jisu Lee, Hor-Gil Hur

**Affiliations:** 1 School of Environment and Energy Engineering, Gwangju Institute of Science and Technology (GIST), Gwangju 61005, Republic of Korea; 2 Department of Physics and Photon Science, Gwangju Institute of Science and Technology (GIST), Gwangju 61005, Republic of Korea; 3 School of Natural Resources and Environmental Science, Department of Biological Environment, Kangwon National University, Chuncheon, Gangwon State 24341, Republic of Korea; 4 Department of Food Biotechnology and Environmental Science, Kangwon National University, Chuncheon, Gangwon State 24341, Republic of Korea; 5 Kimchi Functionality Research Group, World Institute of Kimchi, Gwangju 61755, Republic of Korea

**Keywords:** Vanadium dioxide, biosynthesis, *Shewanella* sp. strain HN-41, nanoparticles, membrane vesicle

## Abstract

Vanadium dioxide (VO_2_) nanoparticles have various application potentials such as smart windows and electronic devices due to their unique phase transition properties. However, conventional VO_2_ synthesis methods require harsh conditions and toxic reducing agents, leading to environmental problems. In this study, we developed an eco-friendly method to biosynthesize VO_2_ nanoparticles using *Shewanella* sp. strain HN-41 under anaerobic conditions at 30°C and neutral pH. Morphological observations revealed that biogenic VO_2_ nanoparticles with an average size of 4.3 nm were in the form of granules presented inside and outside the cells. These nanoparticles were identified as VO_2_ by differential scanning calorimetry (DSC) analysis, which showed a phase transition temperature of 61.9°C, consistent with that of VO_2_. Furthermore, we observed an active formation of vesicles containing VO_2_ nanoparticles by the cross-sectioned transmission electron microscopy (TEM) analysis. Thus, in addition to the direct extracellular formation of VO_2_ nanoparticles through anaerobic respiration, bacterial membrane vesicles likely play a role in expelling nanoparticles from the cell, potentially mitigating their toxicity. These findings highlight metal reducing bacteria could be a biological green agent for the production of valuable VO_2_ nanoparticles under anaerobic environmental conditions.

## Introduction

Vanadium dioxide (VO_2_) has been paid attention as a promising material due to its unique characteristic arising from the reversible phase transition from the rutile phase to the monoclinic phase at 68°C [1]. During the phase transition to the monoclinic phase, electron conductivity decreases by 10^3^-10^4^ times, accompanying the metal-insulator transition, and the length of the rutile crystal structure decreases by ~1% along the c-axis [2, 3]. Owing to these changes, VO_2_ has been extensively studied for applications in thermal sensors and Mott transistors [1]. In addition, VO_2_ is a suitable material for smart windows because it can regulate indoor temperature by changing the transmittance of infrared rays depending on phase transition temperature. For instance, when the indoor temperature is high, VO_2_-based smart windows decrease the temperature by reducing the transmittance of infrared rays from 80% in the rutile phase to 20% in the monoclinic phase. At the low indoor temperature, the transmittance of infrared rays is reverted to 80% by the phase transition to rutile for warming the indoor temperature [4]. For advanced applications, nano-scale VO_2_ materials have been utilized due to their structural advantages such as higher surface areas and infrared ray transmittance compared to bulk materials [3, 5, 6]. Specifically, nanoparticle-based VO_2_ film can solve the problem of low-visible light transmittance in smart windows through its high phase transition efficiency [6].

Despite its high potential for various applications, synthesizing VO_2_ requires harsh conditions and/or toxic reducing agents [7]. For instance, vapor transport methods vaporize bulk vanadium pentoxide (V_2_O_5_) at 700°C under reducing conditions, and then recrystallize to VO_2_ [8]. Although hydrothermal methods address this problem by lowering the synthesis temperature to around 200-300°C [7], these methods require toxic reducing agents including hydrazine and aliphatic alcohol [9, 10]. Considering that current chemical synthetic methods raise potential environmental problems including the consumption of fossil fuels for harsh reaction conditions and the contamination by reducing agents, eco-friendly green methods for VO_2_ synthesis should be developed to solve the drawbacks of recent methods [11].

The biosynthesis of inorganic nanomaterials using metal-dissimilatory bacteria is a green approach coupled with the bioremediation of heavy metals from environments [11-14]. For instance, the bacterial genus *Shewanella* can reduce various inorganic substrates including Fe(III), Mn(IV), As(V), V(V), Cr(VI), U(VI), Se(VI), TC(VII), Te(IV), S^0^, S_2_O_3_, and NO_3_ [11, 15]. The reduced metal ions are precipitated while being synthesized into nanomaterials, such as arsenic-sulfide nanotubes, selenium nanosphere, and iron-oxide nanoparticles, which could be applied as the materials for lithium-ion batteries, photovoltaics, and anti-microbial agents [11, 16-20]. In case of vanadium, many researches have studied on the reduction of aqueous V(V) to V(IV) precipitates using various organisms: bacteria (*Shewanella* strains, *Geobacter metallireducens*, and *Lactococcus raffinolactis*); green algae (*Chlorella sorokiniana* and *Picochlorum oklahomesnsi*); and archaea (*Methanosarcina mazei* and *Methanothermobacter thermautotrophicus*) [12, 13, 21-23]. Although several studies have reported microbial V(V) reduction, the biosynthesis of VO_2_ nanoparticles and its characterization have yet to be reported. Considering the harsh conditions and toxic reducing agents for the typical synthesis of the VO_2_ nanoparticles, the green biosynthesis method under mild conditions is required. In this study, we reported the eco-friendly synthetic method of biogenic VO_2_ nanoparticles through anaerobic respiration of V(V) by *Shewanella* sp. strain HN-41 under neutral pH conditions at 30°C, and secretion mechanism of the nanoparticles via vesicle-mediated transport.

## Materials and Methods

### Culture Conditions for Synthesis of Biogenic VO_2_ Nanoparticles by *Shewanella* sp. Strain HN-41

The culture medium, called *Shewanella* basal medium, was prepared under anaerobic conditions following the protocol by Lee *et al*. [24]. Briefly, one liter of the medium was boiled for 20 min to remove dissolved air, followed by purging with N2 gas (99.9%) for 30 min. Sodium lactate (Junsei, Japan) and V_2_O_5_ (Sigma Aldrich, USA) were added to the medium in the final concentrations of 10 mM and 1 mM, respectively, before autoclaving at 121°C for 15 min. The broth culture of *Shewanella* sp. strain HN-41 incubated in Luria Bertani broth (BD Difco, USA) at 30°C under 180 rpm for 18 h was washed with 30 mM HEPES buffer (pH 7.0; GoldBio, USA) by centrifugation under 3,075 ×*g* for 10 min at 4°C. The washed cells resuspended in *Shewanella* basal medium to a final cell density of OD_600_ = 10.0 were inoculated into the basal medium at the final concentration of 1% (v/v), followed by incubation at 30°C for 5 d. Anaerobic experiments using serum bottles with butyl rubber stoppers and aluminum seals were conducted inside an anaerobic glove box (Coy Laboratory, USA) filled with mixed gas (N_2_: H_2_: CO_2_ = 90: 5: 5).

### Quantification of Vanadium and Lactate Concentrations

An aliquot of 1 ml was periodically sampled using a syringe to quantify vanadium and lactate concentrations in the culture medium. The aliquot was centrifuged under 3,075 ×*g* for 5 min and filtered through a 0.22 μm syringe filter (Advantec, USA). The filtered aliquot of 0.5 ml was serially diluted using 2% (v/v) nitric acid (Chemitop, Republic of Korea). Aqueous vanadium concentration in the culture medium was quantified using iCAP7400DUO inductively coupled plasma optical emission spectroscopy (ICP-OES) (Thermo Fisher Scientific, USA). All nitric acid solutions were preserved in plastic bottles to prevent trace metal contamination from glass materials. Lactate concentration was quantified using high-performance liquid chromatography (HPLC) (Agilent Technology, USA) equipped with SPD-10A UV-Vis detector (Shimadzu, Japan) and RSpak KC-811 column (8.0 mm × 300 mm) (Shodex, Japan). The mobile phase was 5 mM sulfuric acid at a flow rate of 0.5 ml/min for 30 min, and lactate was detected at a wavelength of 210 nm. All experiments were conducted in triplicate.

### Morphological Analysis of Biogenic VO_2_ Nanoparticles

The biogenic VO_2_ nanoparticles synthesized after 5 d incubation were collected by centrifugation under 3,075 ×*g* for 5 min at 4°C. The precipitants were washed three times using anoxic deionized water. The biogenic VO_2_ nanoparticles were separated from bacterial cells using a 0.22 μm syringe filter (Advantec). Morphologies of biogenic VO_2_ nanoparticles were observed using scanning electron microscope (SEM) and transmission electron microscope (TEM). For SEM observation, the washed sample was mounted on silica wafers and dried in an aerobic glove box. The samples were coated with Pt to 20 nm thickness using ion sputter coater (DSR, England). SEM analysis was performed using Veros 5 XHNR SEM (Thermo Fisher Scientific) equipped with Ultim Max 65 energy dispersive X-ray spectroscopy (EDS) (Oxford Instruments, Abingon, England). For TEM observation, the washed sample was mounted on a 200-mesh carbon-coated copper grid and dried in an anaerobic glove box. The analysis was performed using G2 F30 S-Twin TEM (Fei, USA). The elemental composition and crystal structure of the samples were further analyzed using EDS and selected area diffraction pattern (SADP) analysis. The cross-sectioned cell sample was prepared following the modified method of Choi *et al*. [25]. The washed sample was fixed with 2% (v/v) glutaraldehyde and 2% (v/v) paraformaldehyde at room temperature for 4 h. The supernatant was then carefully discarded, and the remaining pellet was washed three times using cacodylate buffer (pH 7.3) by centrifugation under 3,075 ×*g* for 5 min at 4°C. The pellet was dehydrated by a series of ethanol treatments of 30, 50, 70, 90, 95, and 100% (v/v). The dehydrated pellet was embedded in LR white resin (Sigma Aldrich) for 6 h. The resin was then sealed in a gelatin capsule and hardened at 75°C for 24 h. The resin was sectioned to 80-100 μm thickness using a diamond knife. The section was mounted on a nickel grid and subsequently stained with 4% (v/v) uranyl acetate and 2% (v/v) lead citrate.

### Identification of Biogenic VO_2_ Nanoparticles

The washed biogenic VO_2_ nanoparticle samples were lyophilized at -80°C under a 5 mTorr vacuum for 3 d using a TFD8501 freeze-dryer (Ilshinbiobase, Republic of Korea). The lyophilized sample was then analyzed using X-ray diffraction (XRD) and differential scanning calorimetry (DSC). XRD analysis was performed using a SmartLab X-ray diffractometer (Rigaku, Japan) with a scan range of 2θ = 20-40° at a scan speed of 1°/min. For DSC analysis, 5 mg of the sample was loaded in an aluminum concavus pan and sealed using a pierced lid. DSC analysis was conducted using a DSC 204 F1 Phoenix (Netzsch, Germany) with a temperature range from -120°C to 100°C at a heating rate of 10°C/min.

## Results and Discussion

### Changes in Vanadium and Lactate Concentrations

**Fig. 1:**
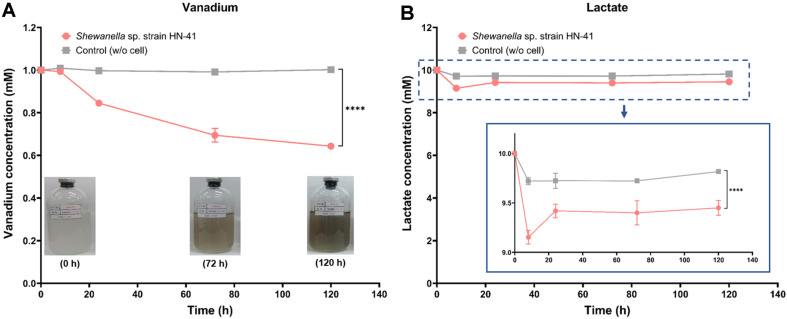
Changes in vanadium (A) and lactate (B) concentrations in the aqueous phase during incubation with *Shewanella* sp. strain HN-41. Digital images of the culture media are shown in panel A, and an enlarged view of the lactate concentration is provided in panel B. Values represent the mean ± standard deviation of triplicate determinations. Statistical significance is indicated by **** (*p* < 0.0001).

The concentrations of aqueous vanadium and lactate in the culture media changed during the incubation with *Shewanella* sp. strain HN-41 (Fig. 1). By day 5, vanadium and lactate were consumed in amounts of 0.36 mM (Fig. 1A) and 0.42 mM (Fig. 1B), respectively. *Shewanella* strains have been reported to be capable of reducing V(V) to V(IV) through anaerobic respiration, using lactate as an electron donor and V(V) as the final electron acceptor [15, 22, 26]. During the incubation, the control sample (without *Shewanella* sp. strain HN-41) remained transparent with no visible change. Otherwise, the dark brown colored precipitants gradually formed in the presence of the strain, suggesting that soluble V(V) was reduced and synthesized to VO_2_ nanoparticles by strain HN-41 (Fig. 1A, inset). In addition, based on Fig. 1, neither vanadium nor lactate concentrations decreased in the control sample, suggesting that no vanadium nanoparticle formation occurred.

### Observation and Identification of Biogenic VO_2_ Nanoparticles

**Fig. 2:**
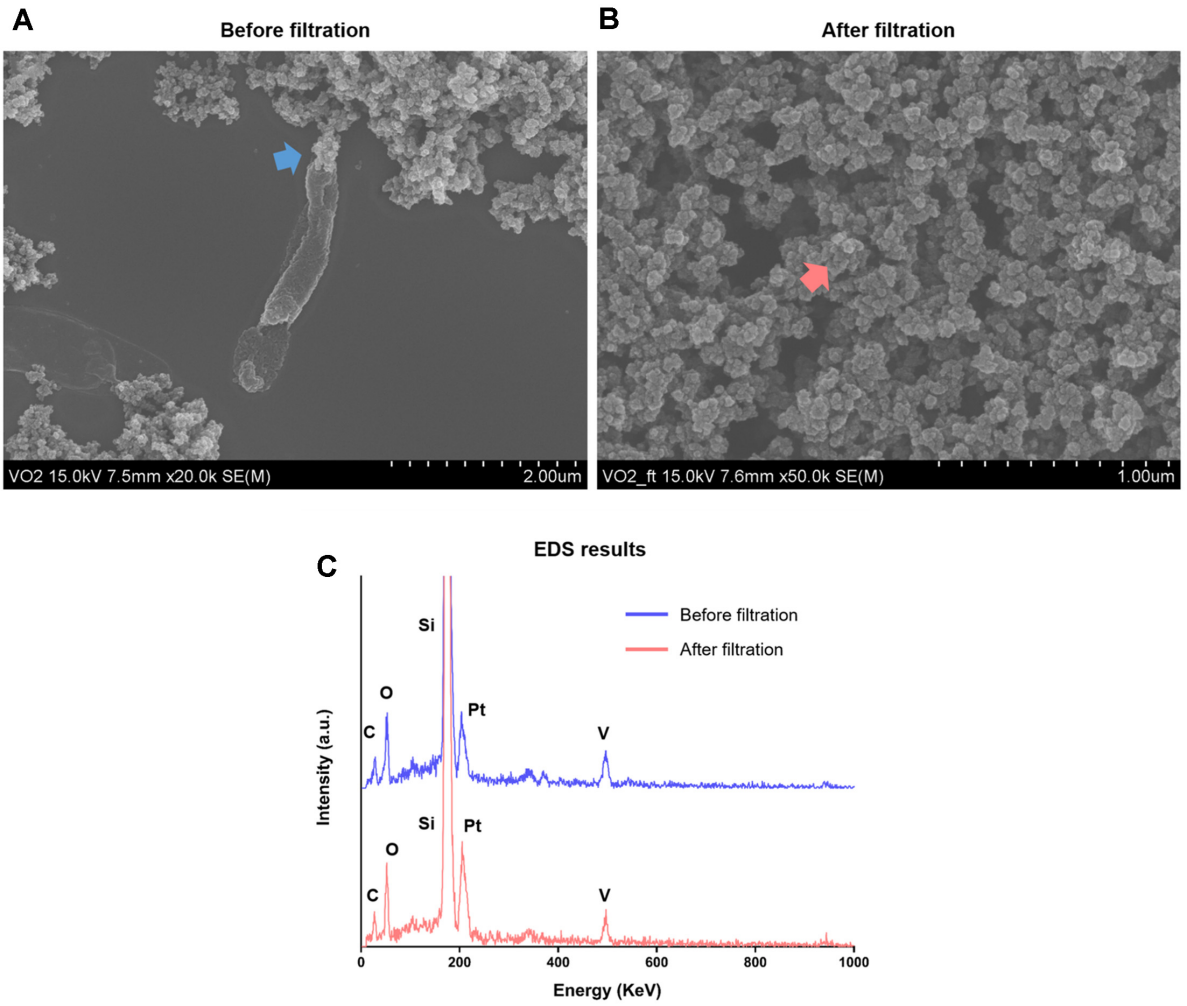
SEM images of biogenic VO_2_ nanoparticles before (A) and after (B) filtration through a 0.22 μm syringe filter, along with their EDS analysis results (C). Blue and red arrows in panel A and B indicate the spots where EDS analysis was performed before and after filtration, respectively.

To observe and identify the dark brown precipitates produced by *Shewanella* sp. strain HN-41, SEM, TEM, XRD, and DSC analyses were performed. SEM-EDS analysis revealed that a number of granules, composed of vanadium, carbon, and oxygen elements, were formed likely secreted by *Shewanella* sp. strain HN-41 (Fig. 2A and 2C). The granules appeared to be secreted out from *Shewanella* sp. strain HN-41 rather than accumulating within the bacterial cells. After filtration, only granules were observed with the absence of bacteria and found to consist of vanadium, carbon, and oxygen elements (Fig. 2B and 2C), indicating that these secreted granules contained biogenic VO_2_ nanoparticles. The peaks of silicon (Si) and platinum (Pt) were detected from the silica wafers on which the samples were mounted and the Pt coating applied to the samples, respectively.

**Fig. 3:**
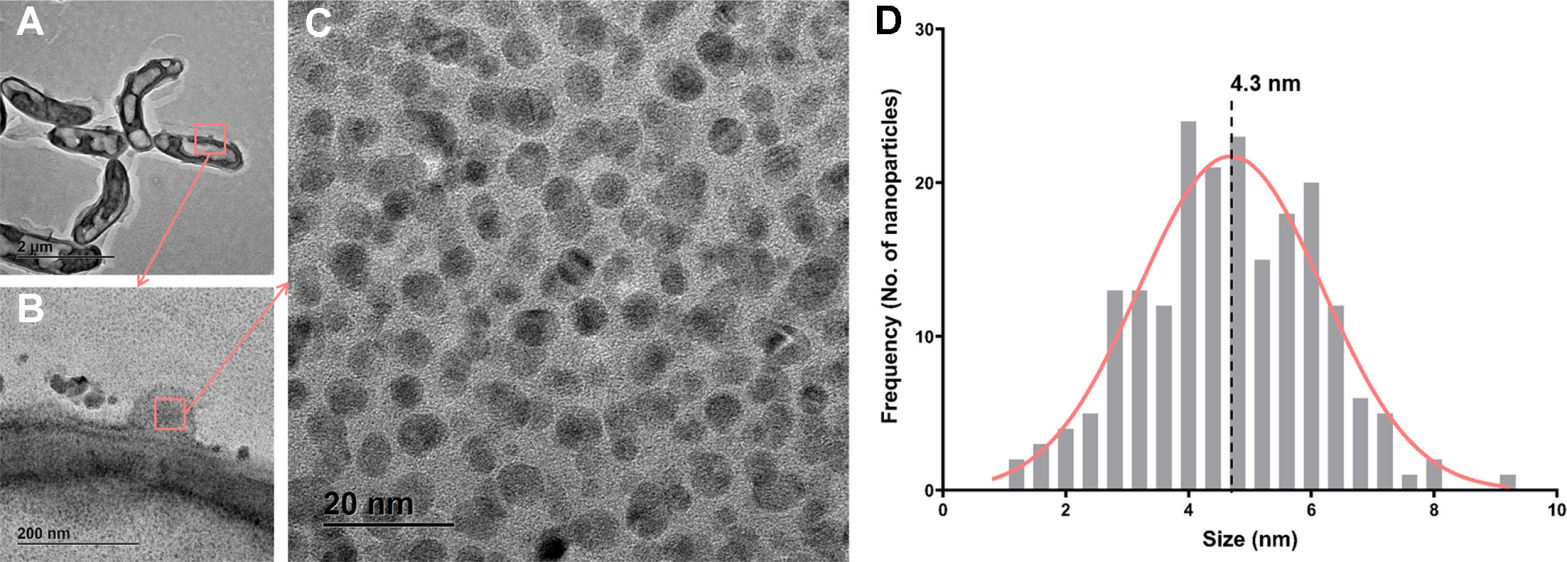
TEM images of biogenic VO_2_ nanoparticles (A-C) and their size distribution. (D). Red squares indicate the enlarged areas shown in the subsequent panels. The size distribution was determined based on the measurement of 200 individual VO_2_ nanoparticles.

To observe the morphologies within the bacterial cells, we further performed TEM analysis. TEM observations revealed the presence of VO_2_ nanoparticles inside and outside of cells. Interestingly, bacterial vesicles were formed on the bacterial surfaces containing nano-sized particles (Fig. 3B and 3C). The average size of the nanoparticles was estimated to be 4.3 nm, as determined from the normal distribution of particle sizes (Fig. 3D). These nanoparticles exhibited lattice crystalline structures when initially exposed to electron beams during TEM observations, while the structures changed and fragmented with prolonged exposure to the beams. Irradiation-induced crystallization/degradation is a common phenomenon seen in TEM observation [27-30]. This phenomenon is known to be more critical in some particular conditions, such as insulators, nanoparticle systems, and oxide systems. Electron beam irradiation onto an insulating system for the TEM method causes charging onto the system surface, and a nanoparticle system with the limited surface to compensate for the effect usually results in a strongly charged surface [27, 28]. Also, for many oxide systems or salt systems, electron beam bombardment and strong charge can cause damage to the crystal structure, since the atomic bonding heavily relies on the oxidation state which is strongly related to the electron-state of each atom [28]. VO_2_ formed in nanoparticles in our experiment is an insulator oxide system in 300K, which makes the system extremely vulnerable under the electron beam. However, the usual electronical applications for VO_2_ systems such as sensors and smart windows do not consider high electron beam dosage, and charging shouldn’t be an issue for well-grounded electronical systems [6].

**Fig. 4:**
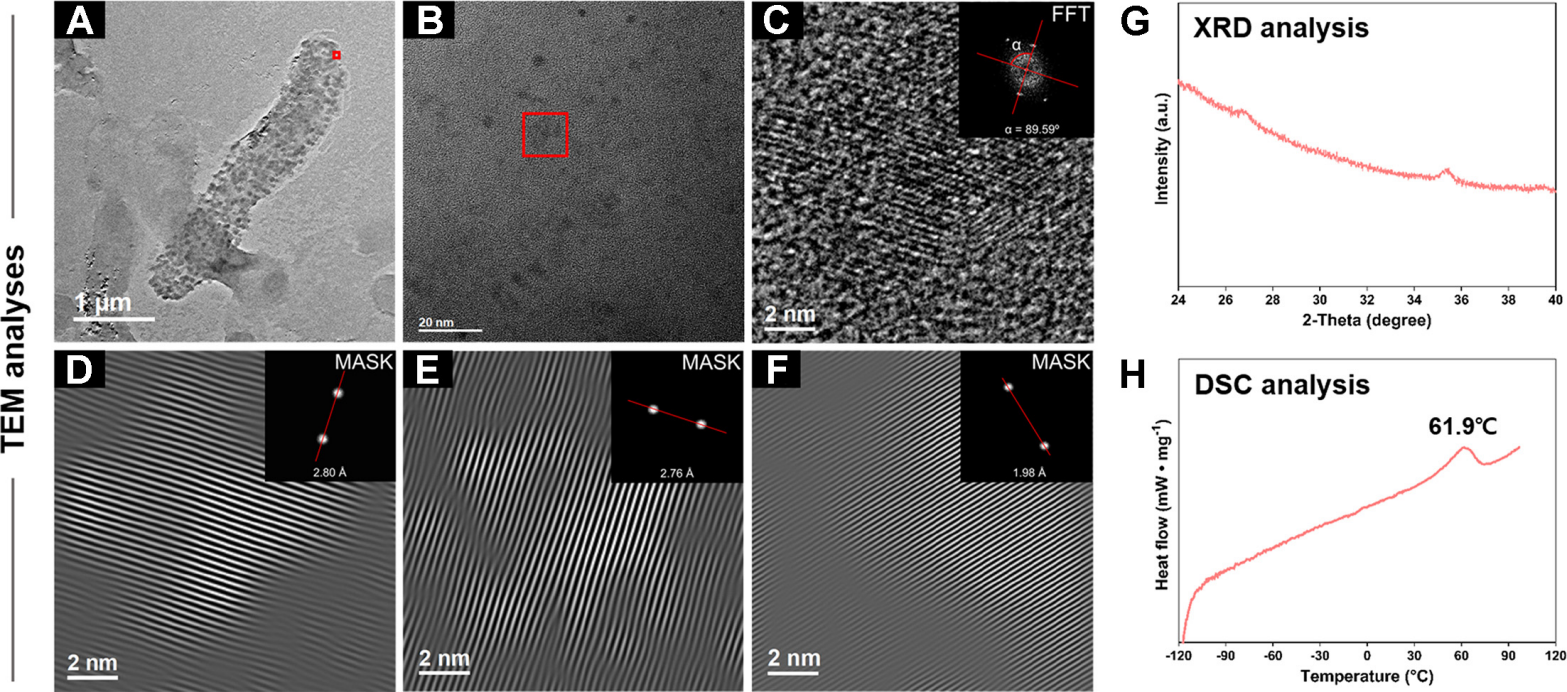
Identification of biogenic VO_2_ nanoparticles through TEM (A-F), XRD (G), and DSC (H) analyses. Red squares in panels A and B indicate the regions of interest (ROI) shown in panel C, cropped for crystalline region analysis. The diffraction pattern and interplanar angle for the crystalline region are shown in the FFT inset (C). Bragg-filtered images generated from IFFT using masked FFT image are presented in panels D, E, and F, with the masked regions indicated in each inset. Interplanar distances corresponding to the three major diffraction signals were calculated from the filtered IFFT images. XRD analysis shows the diffraction pattern of biogenic VO_2_ nanoparticles (G), and DSC analysis shows their phase transition temperature (H).

Using the crystal structure of the nanoparticles from the initial stage, lattice spacing and interplanar angle analyses closely matched with those of V_6_O_13_ (ICDD PDF #01-089-0100, C2/m monoclinic) (Fig. 4A-4F). The interplanar spacing of the lattice structure was 2.80 Å (112), 2.76 Å ($\bar{4}$02) , and 1.98 Å ($\bar{4}$14) , and the interplanar angle between (112) and ($\bar{4}$02) was 89.59º. All values matched the ICDD data for V_6_O_13_ within the 1% error range. However, when the nanoparticles changed and fragmented, the altered crystal structure was unsuitable for material identification. Additionally, XRD analysis also revealed that these nanoparticles were in amorphous phase, making precise identification challenging (Fig. 4G). Therefore, an additional classification method was required to further characterize these nanoparticles.

Vanadium oxides have the unique characteristic of exhibiting a phase transition from insulator to metal at distinct temperatures depending on their types [31, 32]. For instance, VO_2_ exhibits a phase transition at 68°C, close to practical life temperature, while other vanadium oxides such as V_4_O_7_ (-23°C), V_6_O_11_ (-96°C), V_6_O_13_ (-123.1°C), and VO (-147°C) exhibit phase transitions at much lower temperatures [31]. Therefore, characterizing the phase transition temperatures using DSC analysis can be a critical method for identifying the type of vanadium oxide [33, 34]. In this study, DSC analysis revealed that a distinct phase transition of biogenic VO_2_ nanoparticles occurred at 61.9°C, likely consistent with that of VO_2_ at 68°C (Fig. 4H) [31]. The phase transition occurred at the lower temperature could be explained by the stoichiometric impurity and surface strain of nanoparticles [4, 7]. The stoichiometry of VO_2-δ_, or VO_2_ crystal grown in a reduced environment, has been reported to show a wider range of transition temperature [35]. In addition, strain onto VO_2_ crystal is known to change monoclinic-to-rutile transition temperature, from 60°C to 82°C and higher [36]. However, due to lack of the methods to confirm the stoichiometry or the strain inside the nanoparticle, it is limited to provide further confirmation of the phase transition at lower temperatures. Based on measured results, we assumed that the biogenic vanadium nanoparticles produced by *Shewanllea* sp. strain HN-41 are likely to be identified as VO_2_.

**Fig. 5:**
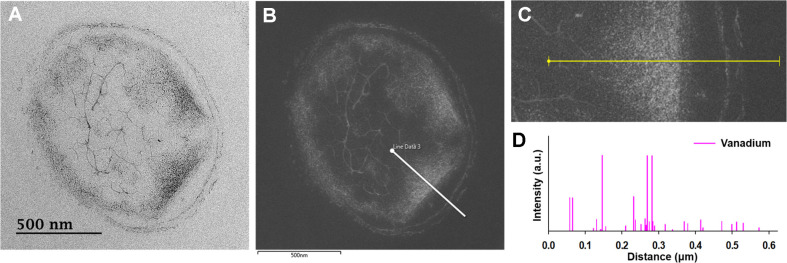
Cross-sectioned TEM images of *Shewanella* sp. strain HN-41 with biogenic VO_2_ nanoparticles (A), line EDS analysis scan sites (B and C), and their EDS spectra of vanadium elements (D). White lines in panel B and yellow lines in panel C represent the EDS scan paths corresponding to the spectra shown in panel D.

These findings highlight a novel approach to synthesize VO_2_ nanoparticles through bacterial reduction systems under mild, environmentally friendly conditions, without the use of harsh chemicals. In general, we assumed that two biological routes for reduction of V(V) to VO_2_ occurred by an anaerobic bacterial respiration on outer membranes and glutathione-mediated reductase and peroxidase enzymatic systems present in the cytoplasm [37-39]. The genes of strain HN-41 encoding these enzymes were identified from whole genome sequence data reported by Kim *et al*. [40]. These biological synthesis pathways could contribute to the presence of the VO_2_ nanoparticles in the cytoplasm and extracellular environments (Figs. 3 and 5).

### Membrane Vesicles Taxing VO_2_ Nanoparticles Formed Inside to Outside Membrane

**Fig. 6:**
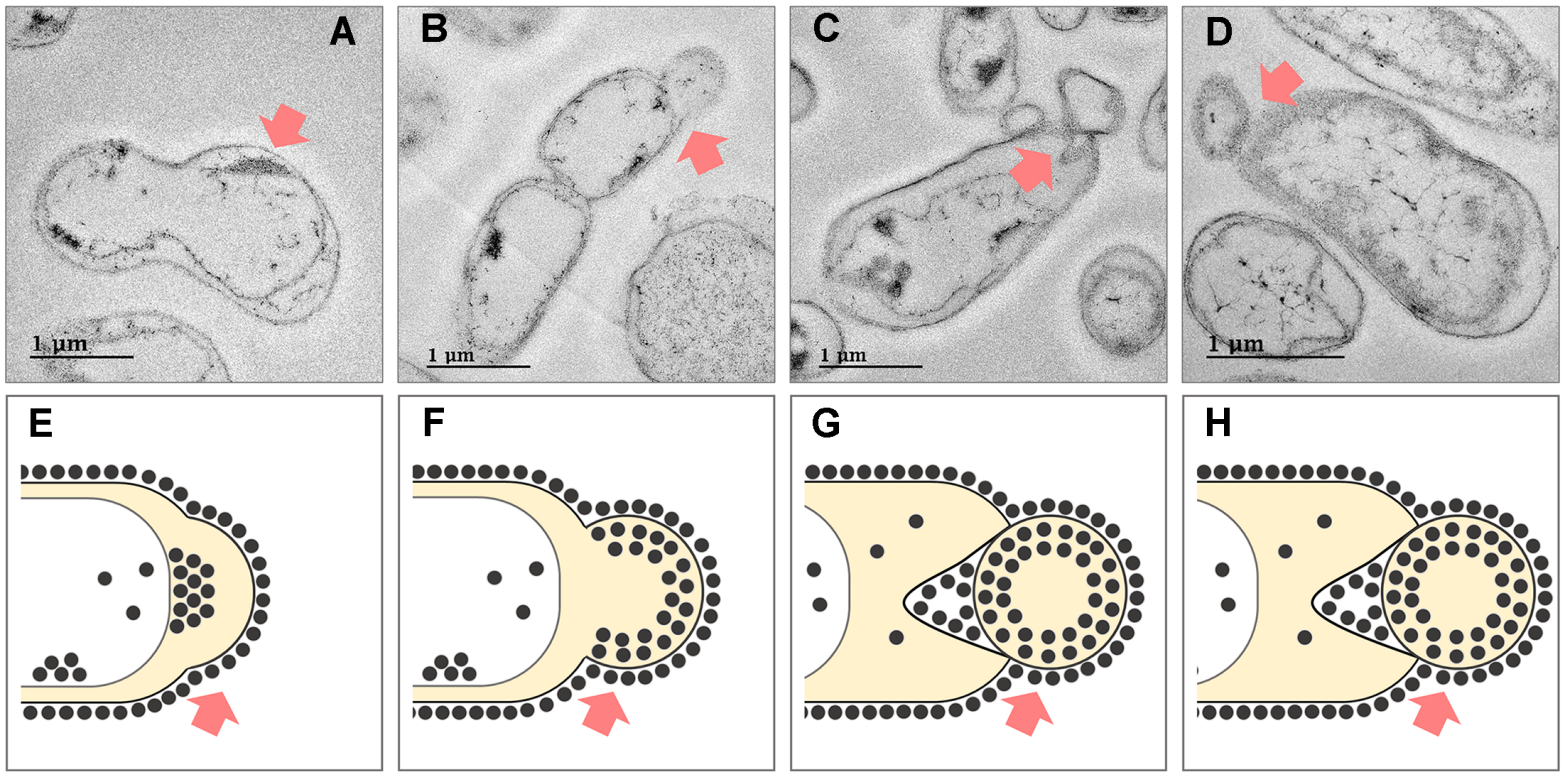
Serial TEM images of cross-sections (A-D) and a schematic diagram illustrating the proposed secretion mechanisms of VO_2_ nanoparticles through vesicle-mediated detoxification in *Shewanella* sp. strain HN-41 (E-H). Biogenic VO_2_ nanoparticles were transferred from the cytoplasm to the periplasm (A and E), and encapsulated in swollen bacterial vesicles (B and F). Then, the vesicles containing the nanoparticles detached from the bacterial outer membrane (C, D, G, and H). Red arrows indicate vesicles containing VO_2_ nanoparticles.

TEM and SEM analyses showed that the vesicles containing the biogenic VO_2_ nanoparticles attached on the bacterial cell surfaces (Fig. 3) were secreted from the cells (Fig. 6). Cross-sectioned TEM analysis (Fig. 5) showed that VO_2_ nanoparticles were densely located in cytoplasm and on the extracellular membranes (Fig. 5A and 5B). EDS analysis confirmed their vanadium composition (Fig. 5B-5D).

To explore the taxing mechanism for the VO_2_ nanoparticles formed in the cytoplasm to the extracellular environments, we further observed additional cross-sectioned TEM images from different areas (Fig. 6). Notably, the series of TEM images illustrated that VO_2_ nanoparticles synthesized in the cytoplasm were encapsulated within the cell membrane (Fig. 6A), which were further developed to form membrane vesicles (Fig. 6A-6C) and eventually detached from the outer membrane (Fig. 6D). These findings suggest that the granules made of the biogenic VO_2_ nanoparticles are likely to be taxed through vesicle-mediated process by *Shewanella* sp. strain HN-41 to the environments (Fig. 6E-6H).

Bacterial membrane vesicles are commonly considered to be involved in heavy metal ion detoxification mechanisms in bacteria [41-44]. However, Budamagunta *et al*. (2023) revealed that *Sporosarcina pasteurii* could respond to heavy metal toxicity by synthesizing nanoparticles, which were subsequently encapsulated within membrane vesicles and secreted [45]. The research suggested that bacterial membrane vesicles were involved not only in heavy metal ion detoxification but also in the secretion of heavy metal nanoparticles.

In previous reports, *Shewanella* strains form the vesicles to enhance the electron transfer chain by connecting the vesicles and cells using nano-wires [46]. Additionally, the vesicles can transport DNA and proteins to adjacent cells [47, 48]. However, the current study showed that *Shewanella* sp. strain HN-41 actively formed membrane vesicles to encapsulate and secrete the nanoparticles after reducing V(V) to VO_2_ and forming the VO_2_ granules. We assumed that this process could relieve toxicity caused by the accumulation of the VO_2_ nanoparticles in the cytoplasm. Thus, the current results provide novel insights into not only understanding vesicle-mediated secretion processes for reducing metal(loid) toxicity but also opening new avenues for biotechnological applications, particularly in the bioremediation of heavy metals and the biosynthesis of nanomaterials.

## Conclusion

This study presents a novel, environmentally sustainable method for synthesizing VO_2_ nanoparticles using *Shewanella* sp. strain HN-41. Considering that traditional VO_2_ synthesis requires harsh reaction environments, including high temperatures and toxic reducing agents, our biosynthetic approach provides an alternative to conventional VO_2_ synthesis, operating under mild anaerobic conditions at 30°C and neutral pH, reducing reliance on harsh chemicals. Overall, this work provides a foundation for advancing eco-friendly biosynthetic methods in nanotechnology and enhances our understanding of microbial processes with potential applications in environmental remediation and biotechnology.
